# Volatile Flavor Profile and Sensory Properties of Vegetable Soybean

**DOI:** 10.3390/molecules27030939

**Published:** 2022-01-29

**Authors:** Luping Guo, Lu Huang, Xi Cheng, Yuan Gao, Xiaoyan Zhang, Xingxing Yuan, Chenchen Xue, Xin Chen

**Affiliations:** 1 Department of Food Science and Engineering, School of Food and Biological Engineering, Jiangsu University, Zhenjiang 212013, China; lp_guo1221@163.com; 2 Institute of Industrial Crops, Jiangsu Academy of Agricultural Sciences, Nanjing 210014, China; huanglu@jaas.ac.cn (L.H.); xyzhang@jaas.ac.cn (X.Z.); yxx@jaas.ac.cn (X.Y.); cx@jaas.ac.cn (X.C.); 3 Department of Food Science and Engineering, Nanjing University of Finance and Economics, Nanjing 210023, China; chengxi628414@163.com; 4 Department of Horticulture, Nanjing Agricultural University, Nanjing 210095, China; 2020804255@stu.njau.edu.cn

**Keywords:** vegetable soybean, volatile flavor profile, sensory properties, electronic tongue, HS-SPME-GC-MS

## Abstract

The volatile flavor profiles and sensory properties of different vegetable soybean varieties popularized and cultivated in China for 20, 10, and 2 years (TW292, X3, and SX6, respectively) were investigated. Nutrient composition analysis revealed that TW292 had a high soluble protein and soluble sugar content but low fat content. The total free amino acid content (15.43 mg/g) and umami free amino acid content (6.08 mg/g) of SX6 were significantly higher (*p* < 0.05) than those of the other varieties. An electronic tongue effectively differentiated between the umami and sweetness characteristics of the vegetable soybeans. Differences in sensory evaluation results were mainly reflected in texture and taste. A total of 41 volatile compounds were identified through HS-SPME-GC-MS, and the main flavor compounds were 1-octen-3-ol, hexanal, (*Z*)-2-heptenal, 2-octene, nonanal, (*Z*)-2-decenal, and 3,5-octadien-2-one. However, the volatile composition of different vegetable soybean varieties exhibited large variability in type and relative contents. Considerable differences in nutritional, organoleptic, and aroma characteristics were found among different varieties. The results of this study will provide a good basis for the assessment and application of the major vegetable soybean varieties grown in China.

## 1. Introduction

Vegetable soybean (*Glycine max* (L.) Merr.), also called ’*maodou*’ in China and ’*edamame*’ in Japan, is a soybean harvested at the R6 growth stage (full pod) when the seeds are still immature and green but are fully developed inside the pods [1,2]. At this stage, the seeds have maximum volume and high levels of sucrose and chlorophyll [2]. Vegetable soybeans have been grown and consumed for almost 1000 years in China [3]. Compared with the grain type soybean harvested after full maturity (R8 stage), vegetable soybeans are large and have unique sensory characteristics [4]. Vegetable soybeans are rich in nutrients, such as carbohydrates, proteins, vitamins, minerals, and phytochemicals [5]. The consumption of vegetable soybeans result in many health benefits, including lowered low-density lipoprotein cholesterol levels and reduced risk of cardiovascular disease [6]. Vegetable soybeans are mainly boiled in the pod and then shelled and consumed as a snack or added to soup or salad [7]. They can also be used as a nutrient supplement or a food ingredient due to their high nutritional value [8]. In addition, vegetable soybeans have higher market value than grain soybeans [9]. As vegetable soybeans are nutrition-rich and have good economic benefits, they play an important role in the food culture of many Asian countries and have gained widespread acceptance in the USA and some European countries within the last 20 years [5,9].

The parameters used in evaluating the quality of vegetable soybeans include flavor, texture, and sensory quality, which are distinct from those used in grading grain soybeans [10]. Green seed coat, large pods, sweet flavor, smooth texture, and distinct seed fragrance are key features of vegetable soybeans [3]. Chemical variability is associated with the organoleptic quality of vegetable soybeans, and chemicals mainly include crude fat, crude protein, soluble sugar, free amino acids, and other compounds [1,11]. Crude fat and crude protein determine the mouthfeel or texture of vegetable soybeans, soluble sugar mainly affects the sweet taste of vegetable soybeans, and sucrose is the predominant sugar. Free amino acids play an important role in the sweetness and umami [7]. Asparagine, alanine, and glutamate are the principal amino acids in vegetable soybeans [7]. In contrast to these compounds, volatile compounds in vegetable soybeans are rarely explored despite the fact that they greatly contribute to flavor diversity [5]. The chemical composition of vegetable soybeans depends on many factors, such as variety, harvest date, and storage conditions [7].

Many varieties of vegetable-type soybeans are cultivated in China, which is the largest producer, consumer, and exporter of vegetable soybeans in the world [12,13]. However, no comprehensive study has reported the volatile composition and organoleptic components of different major vegetable soybean varieties grown in China. Hence, studies on the organoleptic components of different vegetable soybean varieties grown in China may be of great interest. In this study, three vegetable soybean varieties, Taiwan 292 (TW292), Xin 3 (X3), and Suxin 6 (SX6), were selected. TW292 is a variety from Taiwan and has been popularized and grown for more than 20 years. X3 comes from Shanghai and has been popularized for almost 10 years. SX6 was approved by the Crop Breeds Examination and Approval Committee of Jiangsu Province (China) in 2019 and has been popularized over a large area in the last two years. This study was performed to analyze differences in volatile flavor profiles and sensory properties among these three major vegetable soybean varieties. Chemical composition analysis (crude fat, crude protein, soluble protein, soluble sugar, and free amino acids), sensory evaluation, electronic tongue analysis, and SPME-GC-MS were conducted. The aim of this study is to identify the volatile compounds of the major vegetable soybean varieties grown in China and to understand differences in sensory properties among them. The results of this study will provide a good basis for the assessment and application of different vegetable soybean varieties.

## 2. Results and Discussion

### 2.1. Chemical Compositions

The nutrient compositions of TW292, X3, and SX6, particularly crude fat, crude protein, soluble protein, and soluble sugar content, are shown in Table 1. Significant differences in nutrient components were found (*p* < 0.05). The crude fat content of X3 was the highest, whereas that of TW292 was the lowest. Crude protein content had the following sequence from high to low: SX6 > TW292 > X3. TW292 had the highest soluble protein content, and no statistically significant difference was observed between X3 and SX6. X3 had significantly lower soluble sugar content than SX6 and TW292.

Vegetable soybeans of high quality normally have high protein and high soluble sugar content [4,14]. Song et al. reported that the soluble sugar content in vegetable soybeans ranged from 15.131 to 33.979 mg/g [7]. Rao et al. reported that the protein content in 12 vegetable soybeans ranged from 333.2 to 386.0 g/kg [15]. In contrast to previous findings, the contents of soluble sugar and crude protein measured in this study had a large distribution. Fat has a significant impact on the taste quality of vegetable soybeans, and high fat content results in waxy texture. Therefore, with regard to chemical composition, the three vegetable soybeans have their own advantages. TW292 has a high soluble sugar content, which results in sweet taste. X3 has a low soluble sugar content but high fat content, which may result in good texture. SX6 has the highest amount of crude protein content.

### 2.2. Free Amino Acid

**Figure 1:**
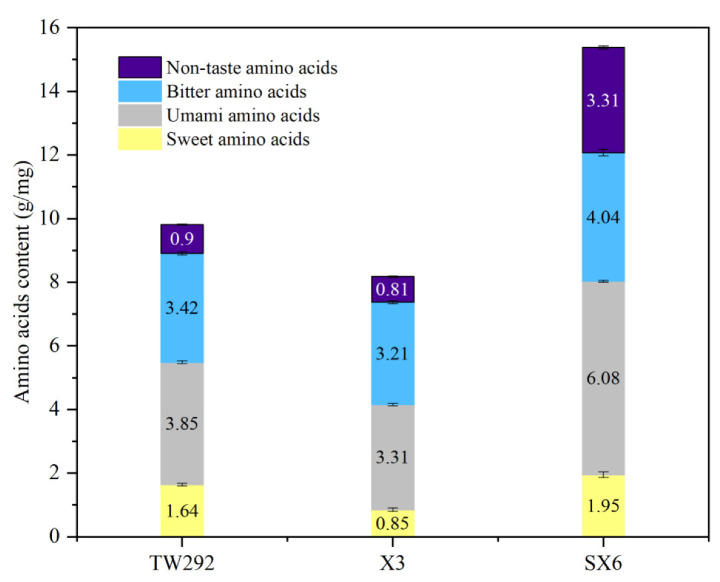
Classification of free amino acid of three vegetable soybean varieties.

Seventeen free amino acids were detected in the vegetable soybeans (Figure 1 and Table 2). Specifically, glutamate, asparagine, and alanine were the major amino acids in the vegetable soybean seeds. The results were similar to those in a previous report [16]. The total free amino acid content of the SX6 variety was 15.43 mg/g, which was significantly higher (*p* < 0.05) than that of X3 (8.21 mg/g) or TW292 (9.84 mg/g). The three varieties contained seven essential amino acids: lysine, phenylalanine, methionine, threonine, isoleucine, leucine, and valine, which accounted for 33.7%, 33.9%, and 32.9% of total amino acid content. The ratio between essential and nonessential amino acids ranged from 0.49 to 0.51, close to the WHO/FAO reference protein model (0.60). Compared with previous findings [7,17], the total free amino acid content in the three varieties was higher. Song et al. reported that the total free amino acid content in eight vegetable soybeans ranged from 4.581 to 10.180 mg/g. It was reported by Flores et al. that the total free amino acid content in three vegetable soybean varieties ranged from 0.49 to 0.71 g/100 g. The amount of free amino acids possibly depended on variety as these vegetable soybeans were cultivated under the same climatic conditions and with the same management factors (irrigation, fertilization, and pest management).

In accordance with the flavor characteristics of free amino acids, the 17 free amino acids were divided into 4 groups: sweet (alanine, glycine, serine, threonine, and proline), bitter (arginine, histidine, isoleucine, leucine, methionine, phenylalanine, and valine), umami (aspartic and glutamic), and tasteless (lysine, tyrosine, and cysteine) [18,19]. SX6 exhibited the highest umami free amino acid content (6.08 mg/g), constituting over 40% of total free amino acids. Sweetness and umami taste are two of the important sensorial attributes of vegetable soybeans [6,17]. The sweet taste comes from its high sugar content, and the umami taste is probably attributed to its amino acid composition [20].

### 2.3. Sensory Properties

Sensory evaluation is currently the main method for estimating the sensory quality of vegetable soybeans. Although people’s senses are effective comprehensive detectors, it’s necessary to recognize that sensory evaluation is easily affected by subjective factors. In this study, sensory evaluation was performed and an electronic tongue was used for the sensory analysis.

#### 2.3.1. Sensory Evaluation

**Figure 2:**
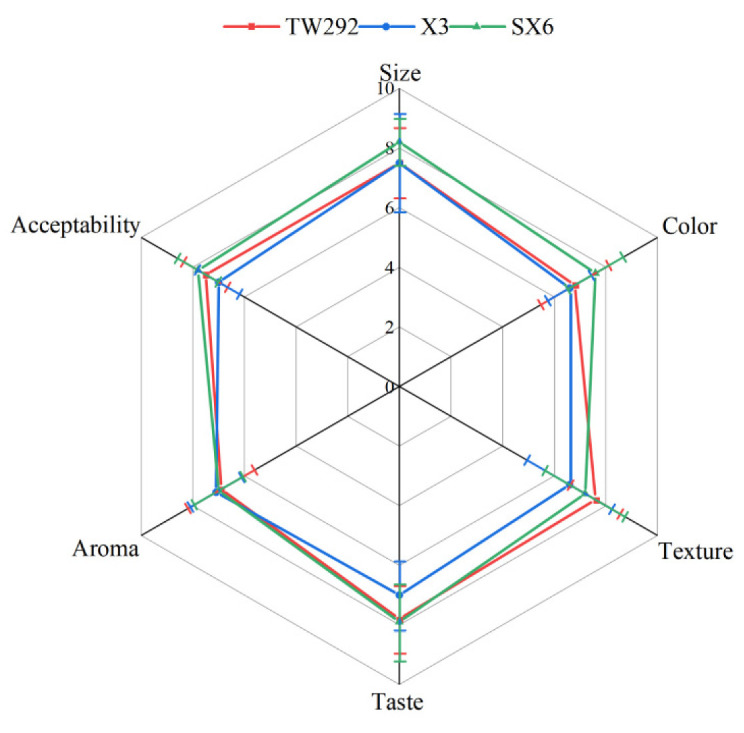
Radar map for sensory evaluation of three vegetable soybeans.

Six attributes, namely, size, color, texture, taste, aroma, and overall acceptability, were used in the sensory evaluation of the three vegetable soybean varieties. The acceptability of all samples was over seven points (“like moderately”), indicating that all the vegetable soybeans are high-quality varieties. However, large variability in sensory evaluation scores was found among the different types of vegetable soybeans (Figure 2). The scores of SX6 for size and color were significantly higher than those of the other varieties. SX6 had a relatively large size and a bright green color. TW292 had the highest score for texture, followed by SX6, and they have a minimal score difference. TW292 had a tender texture, whereas SX6 exhibited a hard texture. In terms of scores for taste, TW292 and SX6 had similar scores, which were significantly higher than X3. With regard to scores for aroma, no statistically significant differences were found among the varieties. It was difficult for the panelists to distinguish aroma among the three varieties. The sequence of the overall acceptability evaluation scores from high to low was as follows: SX6 > TW292 > X3. From the perspective of sensory evaluation, the panelists preferred samples with sweet and umami flavors and tender taste.

Carneiro et al. demonstrated that the appearance, taste, aroma, and texture of vegetable soybeans as sensory attributes significantly affect the acceptability of vegetable soybeans [21]. However, Flores et al. noted that varieties are separated in different factors for flavor and texture, but not appearance [17]. In this study, texture and taste were considered the most important attributes by panelists. TW292 showed the highest soluble sugar content, and SX6 exhibited high soluble sugar and umami amino acid contents, which led to high sensory scores. The results of the sensory evaluation were consistent with Table 1 and Figure 2.

#### 2.3.2. Electronic Tongue

**Figure 3:**
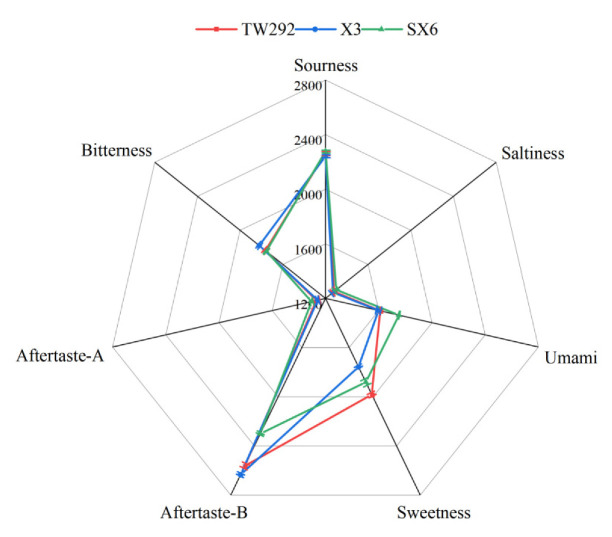
Radar map of the response of electronic tongues of three vegetable soybeans.

Figure 3 was based on the taste sensing scores obtained with an electronic tongue system. Among the five taste response values of the three vegetable soybeans, umami, sweetness, and bitterness showed great differences. In terms of response value for umami, SX6 was 1752.47, which was higher than the values obtained from TW292 and X3 and might be related to the high amino acid content. On the response value for sweetness, TW292 showed the best performance (1981.72), followed by SX6. The electronic tongue data was consistent with the results of the soluble sugar content. In the sensory evaluation, the scores for taste of TW292 and SX6 were significantly higher than the score of X3. The panelists might have had clear preferences for umami and sweetness. The radar chart showed that X3 had the highest response value in bitterness (1820.93). Regarding taste coordination, X3 might be weaker than TW292 and SX6. Consequently, X3 obtained the lowest taste score. The data of the electronic tongue test was greatly correlated with soluble sugar content, free amino acid content, and sensory evaluation score. 

### 2.4. Volatile Compounds

**Figure 4:**
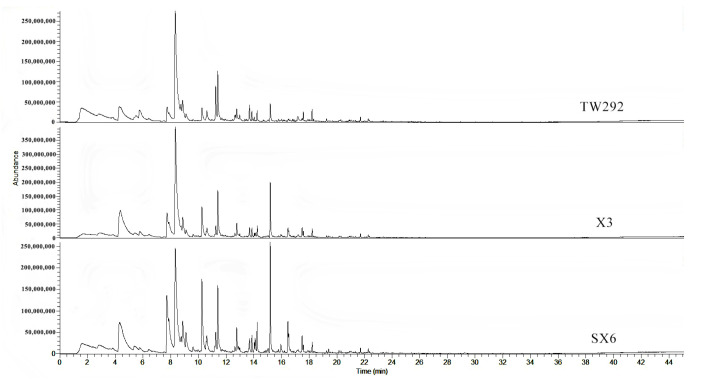
Total ion chromatogram of volatile compounds of vegetable soybeans.

Volatile compounds are associated with aroma and make important contributions to flavor diversity. In this study, the analysis of volatile substances in the three vegetable soybeans was performed using HS-SPME-GC-MS. The total ion chromatograms of the three vegetable soybeans are shown in Figure 4. Differences among the chromatograms were evident, indicating that the volatile composition of the vegetable soybean varieties had large variability. A total of 41 volatile compounds (Table 3) were identified, versus the 27 components detected by Plonjarean et al. in Japanese vegetable soybean ’*Chakaori*’ [22]. They used an acid-phase solvent to extract the aroma compounds and analyzed them through capillary GC-MS. The most abundant flavor compounds that they detected were n–hexanal (0.91%), 1-hexanol (1.79%), 2-hexanal (0.48%), 3-hexene-1-ol (0.49%), and phenylethyl alcohol (0.40%).

**Figure 5:**
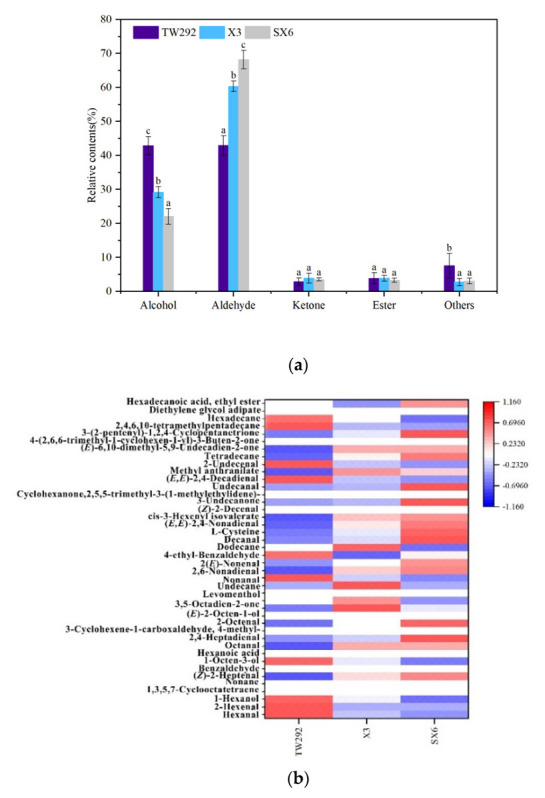
(**a**) Relative content of volatile compounds among different classes in three vegetable soybean samples. Significant difference (*p* < 0.05) is represented by a, b, and c; (**b**) Heat map of the selected major volatile compounds in three vegetable soybeans.

The 41 volatile compounds were classified into 5 groups according to chemical properties: aldehydes (19), alcohols (5), ketones (5), esters (4), and others (8) (Figure 5a). Aldehydes and alcohols were the main volatile substances. A total of 35 kinds of volatile components were detected in TW292, comprising 2 alcohols, 17 aldehydes, 5 ketones, 3 esters, and 8 others. Among them, nonanal, hexanal, and 1-octen-3-ol had a high content (6.97%, 15.01%, and 41.14%, respectively). In X3, 31 volatile components were identified (2 alcohols, 16 aldehydes, 4 ketones, 4 esters, and 5 others). In X3, 2-octenal, (*Z*)-2-decenal, hexanal, (*Z*)-2-heptenal, nonanal, and 1-octen-3-ol were identified as the high-content volatile compounds, which accounted for 6.32%, 7.95%, 8.78%, 9.94%, 10.29%, and 27.80%, respectively. In SX6, 34 volatile components were detected, comprising 5 alcohols, 18 aldehydes, 4 ketones, 2 esters, and 5 others. Nonanal, hexanal, (*Z*)-2-decenal, 2-octenal, (*Z*)-2-heptenal, and 1-octen-3-ol had high relative content (6.88%, 7.41%, 8.84%, 9.67%, 11.70%, and 18.45%, respectively). For the analysis of the aroma characteristics of vegetable soybeans, a heat map based on peak areas was generated for visualization of difference in main aroma compound composition. As shown in Figure 5b, the evident difference in color showed that the volatile composition of the varieties considerably varied in type and relative content. The differences among the proportions of volatile compounds of the varieties led to differences in aroma properties.

Aldehydes are the crucial compounds detected. They are important flavor and fragrance volatiles in many fruits [23]. Hexanal was abundant in the three vegetable soybeans. It contributes a green and grassy off-flavor, which is regarded to be principally responsible for the beany flavor [24]. (*Z*)-2-Heptenal, 2-octene, and (*Z*)-2-decenal were the predominant aldehydes and provided fruity or fat odor notes [25]. Nonanal is naturally found in essential oils, such as rose, citrus, white lemon, and brussels oil, and as a strong oil atmosphere. When diluted, it emits rose and citrus-like aromas. In TW292, hexanal and nonanal had considerably higher relative content than the other aldehydes. Therefore, hexanal and nonanal were identified as the main aldehyde volatile components in TW292. In X3 and SX6, hexanal, (*Z*)-2-heptenal, 2-octene, nonanal, and (*Z*)-2-decenal were the main aldehyde volatiles.

Alcohols constituted the second largest class of identified volatile compounds in the three vegetable soybeans, especially in TW292. Alcohol provides a pleasant aroma (sweet, floral, or fruity) [26]. The most abundant volatile substance detected was 1-octen-3-ol. Its content has a significant impact on the flavor of vegetable soybeans because 1-octen-3-ol has a pleasant loamy aroma and a strong herbal fragrance, similar to the aroma of lavender, rose, and hay. This study showed that 1-octen-3-ol was the main volatile component in vegetable soybeans.

In this study, low levels of ketones, esters, and other compounds were detected in vegetable soybeans and made a low contribution to flavor. The most abundant ketone was 3,5-octadien-2-one. Czerny et al. reported that 3,5-octadien-2-one makes a positive contribution to aroma [27].

On the basis of these results, the volatile composition of three major vegetable soybean varieties in China exhibited a large variability in type. However, 1-octen-3-ol, hexanal, (*Z*)-2-heptenal, 2-octene, nonanal, (*Z*)-2-decenal, and 3,5-octadien-2-one were considered the main flavor compounds. 

## 3. Materials and Methods

### 3.1. Materials

**Figure 6:**
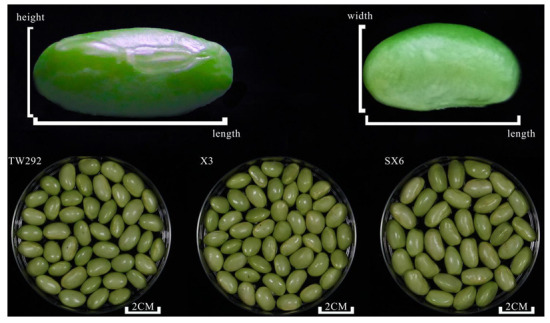
Vegetable soybean samples investigated in this study.

Vegetable soybeans, namely TW292, X3, and SX6, were grown under the same environment in the Liuhe Base of the Jiangsu Academy of Agricultural Sciences (Nanjing, China) situated at 32.08° N 118.40° E. The experiment was planted in a complete block design with three replications with four-row (3.05 m length and 3.66 m width) plots and a row spacing of 0.914 m. Vegetable soybeans were planted on 3 May and harvested at the end of June. All vegetable soybeans were harvested at the R6 stage of seed development (when the majority of the pods were filled before the pods turned yellow and were at the fresh edible stage). Vegetable soybeans of similar maturity without physical injury or infection were selected, washed, and stored until use. According to Song et al. [7], although some of the harvested vegetable soybeans are processed for use in snacks or salads, most are boiled in pods and eaten. Sensory evaluation, electronic tongue analysis, and volatile compound analysis were performed on cooked vegetable soybeans (vegetable soybeans were cooked in boiling water for 5 min). Freeze-dried samples were used in other modes of analysis. Basic information (name and geographical origin) and photographs of these vegetable soybeans are presented in Table 4 and Figure 6. Length, width, height, and hundred-seed weight were used to describe the size of these samples. The hundred-seed weight from high to low was arranged as follows: SX6 (106.619 g) > X3 (73.327 g) > TW292 (64.438 g).

### 3.2. Chemicals and Reagents

Amino acid mix standard solution, bovine serum albumin (BSA), and anthrone were purchased from Sigma-Aldrich Chemical Co. (St. Louis, MO, USA). Solid-phase microextraction (SPME) manual holder and fiber (50/30 µm divinylbenzene/carboxy/polydimethylsiloxane) were purchased from Supelco Co., Ltd. (Bellefonte, PA, USA). All other reagents were of analytical grade and purchased from Sodebio Reagent Co., Ltd. (Nanjing, China).

### 3.3. Chemical Composition Analysis

Freeze-dried vegetable soybean samples were used in the analysis. Soluble sugar content was determined using the anthrone colorimetric method, and glucose was used as the standard [28]. Soluble protein content was measured with the Bradford method, and BSA was used as the standard [29]. Protein content was determined using the Kjeldahl method and a Kjeltec TM2300 autosampler system (Foss Analytical, Hillerd, Denmark). Titration was performed with 0.05 M sulfuric acid and a conversion factor of 6.25 was used to estimate the crude protein [11]. Soxhlet extraction (Extraction System B-811, Buchi, Flawil, Sankt Gallen, Switzerland) with petroleum ether was used in determining crude fat content [30].

### 3.4. Free Amino Acid Analysis

The free amino acid in the vegetable soybean was determined in accordance with the following method [31]: 1 g of freeze-dried vegetable soybean samples was mixed with 3% (*m*/*m*) sulfosalicylic acid and completely homogenized. Then, the homogenate was centrifuged at 10,000× *g* (Eppendorf Centrifuge 5804R, Hamburg, Germany) for 10 min at 4 °C. The solution was filtered through 0.22 μm filters, and the analysis was performed using a Hitachi 8900 amino acid analyzer (Hitachi High-Technologies, Tokyo, Japan). The mixture of FAA standards was used for quantification. Each sample was analyzed in triplicate.

### 3.5. Sensory Evaluation

Sensory assessment was performed using quantitative descriptive analysis (QDA). Panelists composed of 20 women and 20 men were randomly recruited at the Jiangsu Academy of Agricultural Sciences (Nanjing, China). They were 21–58 years old. Before the sensory evaluation, a preliminary test was conducted. The panelists sat at a round table. After the evaluation of the sample, an open discussion was started in order to define the best descriptors for characterizing the samples. Vegetable soybeans were cooked in boiling water for 5 min [17]. After cooling to room temperature, the samples were numbered randomly and fitted in plastic cups. Each panelist received a cup containing the samples and then evaluated the samples with a nine-point hedonic scale (1 = dislike extremely, 2 = dislike very much, 3 = dislike moderately, 4 = dislike slightly, 5 = neither dislike nor like, 6 = like slightly, 7 = like moderately, 8 = like very much, 9 = like extremely). The evaluation included the aspects of size (a high score indicates a large size of vegetable soybean), color (a high score indicates green vegetable soybean), taste (a high score indicates the sweetness and umami of vegetable soybean), aroma (a high score indicates the intense aroma of vegetable soybean), texture (a high score indicates tenderness and waxiness), and overall acceptability (a high score indicates high liking). All the members were provided with mineral water to gargle between evaluations.

### 3.6. Electronic Tongue Analysis

The taste attributes of the vegetable soybean were analyzed using an α-ASTREE electronic tongue (Alpha MOS, Toulouse, France). This taste sensor consists of an array of seven liquid cross-sensitive electrodes, an autosampler, and an associated interface electronic module [32]. The taste sensor output exhibits different patterns for chemical substances that have different taste qualities: bitterness, sourness, saltiness, umami, sweetness, aftertaste-A, and aftertaste-B. Vegetable soybeans (50 g) were cooked in boiling water for 5 min and then ground into juice with three times the amount of distilled water. The juice was extracted for 1 h at 42 °C. After cooling to room temperature, the juice was centrifuged at 10,000× *g* for 10 min to obtain the supernatant, which was aliquoted into a specific electronic tongue beaker. Prior to analysis, the instrument was calibrated and diagnosed in accordance with the instructions of the manufacturer to ensure the stability and reliability of experimental data [33]. Then, the electronic tongue was set for the sample detection method, detection sequence, and cleaning procedures. Sample collection and cleaning were conducted alternately. Deionized water (80 mL) was used to clean the sensors. The instrument parameters were set as follows: delay = 0 s, acquisition time = 180 s, interval = 1 s, and clean time = 10 s. 

### 3.7. Volatile Compound Analysis 

Volatile compounds were extracted and identified through head space solid-phase micro-extraction and gas chromatography coupled with mass spectrometry (HS-SPME-GC-MS). Accurately weighed cooked vegetable soybean (2.0 g) was placed in a 20 mL headspace sample bottle. After water bath extraction at 60 °C for 10 min, the volatiles were sampled with a 50/30 µm DVB/CAR/PDMS fiber purchased from Supelco (Bellefonte, PA, USA) at 60 °C for 40 min [34,35]. Subsequently, the fiber was immediately inserted into the injection port of a Thermo Fisher TSQ8000EVO chromatograph system (Waltham, MA USA) coupled with a quadrupole mass filter and desorbed at 250 °C for 3 min [36]. The extracts were separated on a TG-5MS capillary column (30 m × 0.25 mm I.D., 0.25 µm df). The sample inlet temperature was set at 250 °C and the column temperature was programmed to start at 40 °C. After 2 min, the temperature was ramped to 100 °C at 3 °C/min (held for 1 min), 160 °C at 5 °C/min (held for 1 min), and finally to 280 °C at 10 °C/min (held for 1 min). Helium was used as a carrier gas at a constant flow rate of 1.0 mL/min. The MS conditions were set as follows: EIMS electron energy = 70 eV; ion source temperature = 230 °C; detection was performed in full scan mode over a mass range of 35–800 [37]. Volatile compounds were tentatively identified according to the database of the NIST 2017 library. Retention index (RI) was calculated using *n*-alkanes (C7–C40) as the external reference under the same operating conditions for further confirmation. The relative content of each volatile component was calculated using the peak surface area.

### 3.8. Statistical Analysis

All experiments were run in triplicate, and the results were expressed as mean ± standard deviation. Statistical analysis was performed through one-way ANOVA and Duncan’s multiple test for the identification of significant differences at a level of 0.05. SPSS version 26.0 (SPSS Inc., Chicago, IL, USA) was used. Graphs were drawn using OriginPro 8 (OriginLab Corporation, Northampton, MA, USA).

## 4. Conclusions

The volatile flavor profiles and sensory properties of different vegetable soybean varieties were investigated. Forty-one volatile compounds were identified through HS-SPME-GC-MS and the major flavor components were 1-octen-3-ol, hexanal, (*Z*)-2-heptenal, 2-octene, nonanal, (*Z*)-2-decenal, and 3,5-octadien-2-one. Seventeen free amino acids were detected, and glutamate, asparagine, and alanine were the major amino acids in all varieties. The three varieties contained seven essential amino acids: lysine, phenylalanine, methionine, threonine, isoleucine, leucine, and valine. Differences in sensory evaluation results were mainly reflected in texture and taste. It was difficult for the panelists to distinguish aroma among the three varieties. The electronic tongue effectively differentiated the umami and sweetness characteristics of different vegetable soybeans. Our results indicated that high-quality vegetable soybean varieties tend to have excellent sweet taste and umami taste and tender texture. In terms of chemical components, they contain high soluble sugar content and high sweet and umami amino acid content. Considerable differences in nutritional, organoleptic, and aroma characteristics were found among the varieties. SX6 was found to be a rich source of amino acids and showed strong sweet and umami taste and favorable sensory characteristics. Thus, it was preferred by the panelists. This study provides useful insights into the sensory properties and flavor composition of the major vegetable soybean varieties grown in China. It may be helpful in the potential application of different vegetable soybean varieties as a healthy food.

## Figures and Tables

**Table 1:** Chemical compositions of three vegetable soybean varieties.

Nutrient Content (g/100 g)	TW292	X3	SX6
Crude fat	15.40 ± 0.49 ^a^	22.66 ± 0.23 ^c^	18.71 ± 0.44 ^b^
Crude protein	35.36 ± 0.06 ^a^	37.44 ± 0.23 ^b^	39.35 ± 0.47 ^c^
Soluble protein	11.60 ± 0.10 ^b^	10.29 ± 0.14 ^a^	10.31 ± 0.12 ^a^
Soluble sugar	8.70 ± 0.13 ^c^	3.43 ± 0.10 ^a^	7.03 ± 0.27 ^b^

All values are mean ± standard deviation for triplicate experiments. Values are expressed on a dry matter basis. Significant difference (*p* < 0.05) is represented by ^a^, ^b^, and ^c^.

**Table 2:** Content of free amino acid of three vegetable soybean varieties.

Amino Acid	Flavor Characteristics	Content (mg/g)		
		TW292	X3	SX6
Ala	sweetness	1.15 ± 0.03 ^a^	0.6 ± 0.01 ^b^	1.03 ± 0.05 ^c^
Gly	sweetness	0.15 ± 0.01 ^c^	0.09 ± 0.01 ^a^	0.18 ± 0.02 ^b^
Ser	sweetness	0.09 ± 0.01 ^b^	0.03 ± 0.02 ^a^	0.52 ± 0.04 ^c^
Thr	sweetness	0.16 ± 0.03 ^b^	0.05 ± 0.01 ^a^	0.10 ± 0.02 ^c^
Pro	sweetness	0.09 ± 0.01 ^b^	0.07 ± 0.02 ^a^	0.12 ± 0.01 ^b^
Asp	umami	1.06 ± 0.04 ^c^	1.25 ± 0.02 ^a^	1.35 ± 0.04 ^b^
Glu	umami	2.79 ± 0.05 ^a^	2.07 ± 0.05 ^a^	4.73 ± 0.05 ^b^
Arg	bitterness	1.52 ± 0.02 ^a^	1.97 ± 0.03 ^a^	2.07 ± 0.13 ^b^
His	bitterness	1.52 ± 0.04 ^a^	0.83 ± 0.03 ^a^	1.66 ± 0.03 ^b^
Iss	bitterness	0.05 ± 0.01 ^a^	0.08 ± 0.01 ^b^	0.09 ± 0.01 ^b^
Leu	bitterness	0.08 ± 0.01 ^a^	0.13 ± 0.02 ^b^	0.08 ± 0.01 ^a^
Met	bitterness	0.03 ± 0.01 ^a^	0.03 ± 0.01 ^b^	0.06 ± 0.01 ^c^
Phe	bitterness	0.13 ± 0.01 ^c^	0.10 ± 0.02 ^b^	0.01 ± 0.01 ^a^
Val	bitterness	0.12 ± 0.02 ^b^	0.09 ± 0.02 ^a^	0.14 ± 0.02 ^c^
Tyr	non-taste	0.31 ± 0.02 ^b^	0.36 ± 0.02 ^a^	2.55 ± 0.04 ^c^
Cys	non-taste	0.03 ± 0.01 ^a^	0.02 ± 0 ^b^	0.05 ± 0.01 ^b^
Lys	non-taste	0.56 ± 0.01 ^b^	0.43 ± 0.01 ^a^	0.70 ± 0.02 ^c^
Total FAAs		9.84 ± 0.09 ^b^	8.21 ± 0.06 ^a^	15.43 ± 0.04 ^c^

All values are mean ± standard deviation for triplicate experiments. Values are expressed on a dry matter basis. Significant difference (*p* < 0.05) is represented by ^a^, ^b^, and ^c^.

**Table 3:** Relative content of volatile compounds among different classes in vegetable soybean samples.

Number	RI	Compounds	Formula	Relative Contents (%)		
				TW292	X3	SX6
1	802	Hexanal	C_6_H_12_O	15.01	8.78	7.41
2	858	2-Hexenal	C_6_H_10_O	1.54	1.1	1.04
3	875	1-Hexanol	C_6_H_14_O	1.53	1.26	0.99
4	892	1,3,5,7-Cyclooctatetraene	C_8_H_8_	1.28	ND	ND
5	906	Nonane	C_9_H_20_	ND	0.93	ND
6	963	(*Z*)-2-Heptenal	C_7_H_12_O	4.02	9.94	11.7
7	967	Benzaldehyde	C_7_H_6_O	ND	ND	4.58
8	984	1-Octen-3-ol	C_8_H_16_O	41.14	27.8	18.45
9	1004	Hexanoic acid	C_6_H_12_O_2_	ND	ND	0.93
10	1008	Octanal	C_8_H_16_O	1.55	3.89	3.76
11	1018	2,4-Heptadienal	C_7_H_10_O	1.26	1.61	2.77
12	1040	3-Cyclohexene-1-carboxaldehyde, 4-methyl-	C_8_H_12_O	ND	ND	0.78
13	1064	2-Octenal	C_8_H_14_O	3.15	6.32	9.67
14	1076	(*E*)-2-Octen-1-ol	C_8_H_16_O	ND	ND	0.8
15	1080	3,5-Octadien-2-one	C_8_H_12_O	2.37	3.15	2.57
16	1100	Levomenthol	C_10_H_20_O	ND	1.17	0.75
17	1104	Undecane	C_11_H_24_	2.18	ND	ND
18	1109	Nonanal	C_9_H_18_O	6.97	10.29	6.88
19	1160	2,6-Nonadienal	C_9_H_14_O	0.84	0.6	0.5
20	1167	2(*E*)--Nonenal	C_9_H_16_O	1.42	2.24	2.43
21	1172	4-ethyl-Benzaldehyde	C_9_H_10_O	0.36	ND	0.57
22	1202	Dodecane	C_12_H_26_	1.21	1.03	1.11
23	1205	Decanal	C_10_H_20_O	1.58	1.92	1.27
24	1220	L-Cysteine	C_3_H_7_NO_2_S	0.38	0.45	0.6
25	1222	(*E*,*E*)-2,4-Nonadienal	C_9_H_14_O	0.2	0.7	1.52
26	1228	cis-3-Hexenyl isovalerate	C_11_H_20_O_2_	1.02	1.95	2.41
27	1269	(*Z*)-2-Decenal	C_10_H_18_O	2.28	7.95	8.84
28	1295	3-Undecanone	C_11_H_22_O	0.14	ND	ND
29	1301	Cyclohexanone,2,5,5-trimethyl-3-(1-methylethylidene)-	C_12_H_20_O	0.52	0.56	0.82
30	1314	Undecanal	C_11_H_22_O	0.26	ND	ND
31	1325	(*E*,*E*)-2,4-Decadienal	C_10_H_16_O	1.73	1.19	2.38
32	1357	Methyl anthranilate	C_8_H_9_NO_2_	2.47	0.72	0.41
33	1372	2-Undecenal	C_11_H_20_O	0.64	1.48	1.27
34	1405	Tetradecane	C_14_H_30_	0.61	0.4	0.34
35	1462	(*E*)-6,10-dimethyl-5,9-Undecadien-2-one	C_13_H_22_O	0.2	0.33	0.4
36	1493	4-(2,6,6-trimethyl-1-cyclohexen-1-yl)-3-Buten-2-one	C_13_H_20_O	0.13	0.17	0.16
37	1538	3-(2-pentenyl)-1,2,4-Cyclopentanetrione	C_10_H_12_O_3_	0.2	0.25	0.34
38	1576	2,4,6,10-tetramethylpentadecane	C_19_H_40_	0.28	0.18	0.16
39	1606	Hexadecane	C_19_H_40_	0.32	0.3	0.25
40	1651	Diethylene glycol adipate	C_10_H_18_O_6_	0.24	ND	ND
41	2000	Hexadecanoic acid, ethyl ester	C_18_H_36_O_2_	ND	0.14	0.21

ND: not detected.

**Table 4:** Name, geographical origin, and agronomic traits of vegetable soybean samples investigated in this study.

Name	Place of Origin	Length/cm	Width/cm	Height/cm	Hundred-Grain Weight/g
TW292	Taiwan	1.5	1.0	0.8	64.438
X3	Shanghai	1.5	1.1	0.8	73.327
SX6	Jiangsu	1.5	1.2	1.0	106.619

The length and width are defined in Figure 6.

## Data Availability

All data are reported in this manuscript.

## Sample Availability

Samples of the compounds are available from the authors.
